# Improving Experience of Psychotherapy Training for Core Psychiatry Resident Doctors in North Central London

**DOI:** 10.1192/bjo.2026.11332

**Published:** 2026-06-30

**Authors:** Giedre Cesnaite, Marion Neffgen, Thomas Hillen

**Affiliations:** North London NHS Foundation Trust, London, United Kingdom

## Abstract

**Aims:**

Undertaking a psychotherapy long case is part of the Core Psychiatry Training curriculum. While the value of psychological skills within psychiatry is well recognised, for many trainees starting the first psychotherapy case is an anxiety provoking task. The need for more formal teaching has been identified in previous studies. The aim of the project was to understand challenges faced by resident doctors and based on this knowledge, to improve the quality of psychotherapy training in North Central London (NCL).

**Methods:**

NCL doctors in Core and Higher Psychiatry Training were invited to complete an anonymous survey which consisted of qualitative and quantitative questions. The feedback was used to design 2 three-hour long teaching sessions between September and October 2025. Both modules had strong focus on experiential learning which included reflecting on video recorded psychotherapy sessions and discussing observations in small groups, whilst providing only brief theoretical introduction to core psychodynamic concepts. The overall experience and effectiveness of teaching was evaluated by collecting data through formal and informal feedback.

**Results:**

A total of 14 responses to the initial survey were received. 50% of respondents hadalready completed a long case, 29% were seeing a patient, 21% were preparing to start by attending a supervision group.

Qualitative analysis showed themes of being underprepared and having not enough formal teaching before seeing a long case. Doctors who were about to start the long case wanted more theoretical teaching, while the ones who had already completed the long case put emphasis on the value of learning through experience e.g. in Balint and supervision groups. All trainees who had already started or completed the long case found the experience very useful or useful (73% and 27% respectively) in helping them to understand psychodynamic concepts better.

Psychotherapy teaching modules were very well attended. First and second teaching session formal feedback was overwhelmingly positive with 40 and 32 responses rating teaching as good (15% and 28%) or excellent (83% and 72%), respectively. Respondents particularly emphasised the usefulness of interactive elements of the teaching, seeing psychotherapy in action through the video material and the discussions in small groups.

**Conclusion:**

While Core Psychiatry Training curriculum provides little guidance on provision of psychotherapy teaching, our project highlighted the usefulness of interactive and experiential teaching methods, which was evidenced by the initial survey results, high teaching attendance rates and very positive formal and informal feedback.

